# Altered Processing of Visual Stimuli in Vestibular Migraine Patients Between Attacks: A Combined VEP and sLORETA Study

**DOI:** 10.3389/fnhum.2021.762970

**Published:** 2021-12-24

**Authors:** Jiahao Liu, Qi Zhang, Maojin Liang, Yajing Wang, Yuebo Chen, Junbo Wang, Jiahong Li, Ling Chen, Leyin Yu, Yinglin Cai, Yiqing Zheng, Yongkang Ou

**Affiliations:** ^1^Department of Otolaryngology, Sun Yat-sen Memorial Hospital, Sun Yat-sen University, Guangzhou, China; ^2^Institute of Hearing and Speech-Language Sciences, Sun Yat-sen University, Guangzhou, China; ^3^School of Foreign Languages, Shenzhen University, Shenzhen, China; ^4^Department of Hearing and Speech Science, Guangzhou Xinhua College, Guangzhou, China

**Keywords:** vestibular migraine, visual evoked potential, sLORETA, cortex abnormalities, neural mechanism

## Abstract

**Objective**: Vestibular migraine (VM) is one of the most common causes of recurrent vertigo, but the neural mechanisms that mediate such symptoms remain unknown. Since visual symptoms and photophobia are common clinical features of VM patients, we hypothesized that VM patients have abnormally sensitive low-level visual processing capabilities. This study aimed to investigate cortex abnormalities in VM patients using visual evoked potential (VEP) and standardized low-resolution brain electromagnetic tomography (sLORETA) analysis.

**Methods**: We employed visual stimuli consisting of reversing displays of circular checkerboard patterns to examine “low-level” visual processes. Thirty-three females with VM and 20 healthy control (HC) females underwent VEP testing. VEP components and sLORETA were analyzed.

**Results**: Patients with VM showed significantly lower amplitude and decreased latency of P1 activation compared with HC subjects. Further topographic mapping analysis revealed a group difference in the occipital area around P1 latency. sLORETA analysis was performed in the time frame of the P1 component and showed significantly less activity (deactivation) in VM patients in the frontal, parietal, temporal, limbic, and occipital lobes, as well as sub-lobar regions. The maximum current density difference was in the postcentral gyrus of the parietal lobe. P1 source density differences between HC subjects and VM patients overlapped with the vestibular cortical fields.

**Conclusion**: The significantly abnormal response to visual stimuli indicates altered processing in VM patients. These findings suggest that abnormalities in vestibular cortical fields might be a pathophysiological mechanism of VM.

## Introduction

Vestibular migraine (VM) is one of the most common conditions contributing to recurrent vertigo and afflicts up to 1% of the whole population with female predominance (Russo et al., 2014). Typical signs of VM include a heightened sensitivity to head motion or visual stimuli, spatial misperceptions, and/or sudden feelings of lopsidedness or tilt. These symptoms often severely limit daily functioning in VM patients. Different from typically brief migraine auras, VM symptoms can last from hours to several days (Beh, 2019). Despite well-defined diagnostic criteria, like those proposed by the Bárány Society and the Migraine Classification Subcommittee of the International Headache Society (Lempert et al., 2012), VM pathophysiology is still unclear and remains controversial. While it is agreed that genetic, epigenetic, and environmental factors are probably involved in VM progression, there is an ongoing dispute concerning whether its primary origin is central or peripheral (Espinosa-Sanchez and Lopez-Escamez, 2015).

Modern neuroimaging methods have been used to observe how neural pathways work in subjects with VM, with a particular focus on the multisensory integration network. Functional neuroimaging showed dysmodulation attributed to vestibulo-thalamo-cortical dysfunction—the pathogenic mechanism underlying VM—in the multimodal sensory integration and processing of vestibular and nociceptive information (Espinosa-Sanchez and Lopez-Escamez, 2015). In 2016, Teggi et al. (2016) reported enhanced responses of multimodal association brain areas [Brodmann area (BA) 40, BA 31/5] and reduced activation of occipital regions in VM patients. In 2017, Messina et al. (2017) indicated that unusual brain sensitization might result in dismodulation of multimodal sensory integration and processing cortical areas in VM patients. As visual symptoms and photophobia are common clinical features of VM, we hypothesized that patients with VM have abnormally sensitive low-level visual processing capabilities. A primary approach to analyzing visual processing integrity is the use of visual evoked potentials (VEPs; Sulejmanpasic and Drnda, 2017). Most of the above studies focused on brain activation using functional magnetic resonance imaging with high spatial resolution but relatively poor temporal resolution. We pay particular attention to the temporal dimensions of these abnormalities, which seem crucial to understanding functional brain changes in VM patients and their clinical correlations. The temporal precision, low cost, and noninvasiveness of VEP measurement make it particularly well suited to study functional brain changes associated with VM (de Tommaso et al., 2014). We specifically selected patients between VM attacks because altered brain metabolism has been found, and patients show activation of the bilateral cerebellum and frontal cortices and deactivation of the bilateral posterior parietal and occipitotemporal areas during VM attacks (Shin et al., 2014).

Vestibular symptoms are also extremely common during the interictal period (Beh et al., 2019), likely because vestibular-sensitive neurons respond to a range of modalities (Grusser et al., 1990a, b; Vuralli et al., 2018). No primary vestibular cortex has been identified, and vestibular signals are generally conveyed to the cerebral cortex (Guldin and Grusser, 1998; Vuralli et al., 2018). Vestibular information processing involves polymodal association areas in the parietal, temporal, and insular cortices and cortical areas associated with spatial orientation (e.g., primary somatosensory cortex, primary visual cortex; Vuralli et al., 2018).

The existing evidence suggests that the central nervous system is altered in VM patients. The aim of the present study was to compare VEP responses between patients with VM patients and healthy control (HC) subjects. We hypothesized that cortex responses activated by visual stimuli would be different between groups. To test this hypothesis, we used parametrically modulated reversing checkerboard images to examine how the physical property of luminance affects early VEPs (i.e., initial stages of visual processing that are strongly influenced by physical stimulus properties). A previous study demonstrated larger VEP responses for higher luminance levels in the visual stimuli (Johannes et al., 1995), leading to our prediction of parametric modulation of early VEP components as a function of overall luminance in the checkerboard images. Moreover, we expected a functional difference between VM patients and HC subjects already at early processing levels in the visual hierarchy. Thus, we applied electroencephalography (EEG)-standardized low-resolution brain electromagnetic tomography (sLORETA; Pascual-Marqui, 2002) to analyze cortex activity in response to visual stimulation. We employed the VEP and sLORETA differences between VM patients and HC subjects to identify disordered cortical activities associated with VM. We hypothesized that VM patients would show decreased P1 amplitudes and lower P1 neural activities in VM-related brain regions.

## Materials and Methods

### Participants

Thirty-three female patients (30 without aura and three with aura), diagnosed as typical VM according to previously described criteria [Lempert et al., 2012; Headache Classification Committee of the International Headache Society (IHS), 2013] were prospectively recruited from the population seen at the Department of Otolaryngology, Sun Yat-Sen Memorial Hospital, Sun Yat-Sen University. All patients were clinically evaluated and diagnosed by the same otolaryngologist after excluding other etiologies which could cause recurrent vertigo attacks, and all patients underwent VEP recordings on days 2–10 after a VM attack, they were attack-free at least 12 h before and after the recordings. Peripheral vestibular dysfunction was found in 7 of 33 VM patients on videonystagmography (VNG) recordings. Furthermore, they did not have any way to prevent migraine, vertigo, or dizziness, and no topiramate, magnesium, or other vestibular inhibitor drugs were allowed during the preceding 1 month. Twenty female subjects with no family history of migraine and no history of chronic pain, substance abuse, or neurologic, psychiatric, or systemic disorders were recruited as HC subjects. There were no significant differences in age between groups. All participants were right-handed with normal or corrected-to-normal visual acuity. Patients with VM, and healthy controls did not report other neurological, psychiatric, audiovestibular, diabetes mellitus, hypertension, vascular/heart diseases, hyper-cholesterolaemia, or other major systemic disorders. Moreover, participants who abused alcohol, nicotine, or other substances were excluded. The clinical and demographic characteristics of the VM and HC groups are summarized in Table 1.

**Table 1:** Demographic and clinical characteristics of patients.

Characteristics	VM (n = 33) Mean ± SD	HC (n = 20) Mean ± SD
Sex	Female	Female
Age	44.55 ± 13.70	43.80 ± 11.85
VM disease duration (years)	6.20 ± 6.28	
Migraine disease duration (years)	10.67 ± 6.22	
Attack frequency per month	2.74 ± 5.25	

HC, healthy control; VM, vestibular migraine.

### Visual Stimuli

We adopted visual stimuli consisting of reversing displays of circular checkerboard patterns reported by Sandmann and colleagues (Sandmann et al., 2012), which have been used to examine cross-modal reorganization in the auditory cortex of cochlear implant users. There were four different pairs of patterns that varied in terms of luminance ratio. The proportions of white pixels in the stimulus patterns were 12.5% (Level 1), 25% (Level 2), 37.5% (Level 3), and 50% (Level 4). The contrast between white and black pixels was identical in all stimuli.

Subjects were comfortably seated in front of a high-resolution 19-inch VGA computer monitor at a viewing distance of approximately 1 m in a soundproof and electromagnetically shielded room. All stimuli were presented via E-prime 2.0 stimulus software that is compatible with Net Station 4 (Electrical Geodesics, Inc.). The checkerboard stimulus remained on the monitor for 500 ms and was immediately followed by blank-screen inter-stimulus intervals that also lasted for 500 ms. Each presented blank stimulus image included a fixation point (a white cross) on the center of the screen. Participants performed four experimental blocks (i.e., conditions) in which they were presented with one of the four image pairs. The block order was counterbalanced across participants. In the course of the experimental session, each checkerboard image was repeated 60 times, resulting in a total of 480 stimuli (four conditions, two images, 60 repetitions). Participants were instructed to keep their eyes on the pictures before each condition and were allowed to have rest for 1 min between blocks.

### EEG Recording and Analyses

EEG data were continuously recorded by a 128-Channel Dense Array EEG System with Hydrogel Geodesic Sensor Nets (EGI, USA). The sampling rate was 1 kHz, and electrode impedances were kept below 50 kΩ. For ERP analyses, individual participant data were band-pass filtered offline at 0.3–30 Hz and segmented with 100-ms pre-stimulus and 600-ms post-stimulus times. Artifact rejection set at 200 mV was applied to visual EEG signals, and epochs were rejected if they contained any eye blinking (eye channel > 140 mV) or eye movement (eye channel > 55 mV). Bad channels were removed from the recording. Data were then re-referenced using a common average reference. The data were baseline corrected to the pre-stimulus time of −100 to 0 ms.

Amplitudes and latencies of the P1-N1-P2 complex on the 75(Oz) electrode for individual participants were analyzed. The time frames for the P1, N1, and P2 were set between 50 and 120 ms, 100 and 170 ms, and 200 and 290 ms, respectively. The amplitudes of the P1, N1, and P2 peaks were measured from the baselines to the peak values. Individual subject latencies were defined at the highest peak amplitude for each VEP component. Individual waveform averages were averaged together for each group to compute a grand-average waveform. We compared the amplitudes and latencies of the VEP components between groups with respect to the four checkerboard images.

For statistical analysis, VEP components were subjected to separate repeated-measures analyses of variance (ANOVAs) with condition (Levels 1–4) as the within-subject factor and group (VM patients and HC subjects) as the between-subjects factor. Significant main effects and interactions (*p* < 0.05) were followed-up with *post hoc* t-tests, and Greenhouse–Geisser corrections were applied when the sphericity assumption was violated. Topographical displays are based on the whole scalp region.

### sLORETA

ERP source analyses were performed using a standardized head model to estimate the intracerebral sources on the basis of an sLORETA algorithm (Pascual-Marqui, 2002), which is a functional imaging method based on electrophysiological and neuroanatomical constraints. This method offers precise localization (Wagner et al., 2004; Sekihara et al., 2005) but low spatial resolution. sLORETA software was provided online by the KEY Institute for Brain-Mind Research, Zurich, Switzerland^1^. Source analyses were only performed in the time frame of the P1 component since scalp-recorded potentials revealed systematic differences between groups specifically for P1 latency. The latency (mean ± SD) from the Oz electrode in each group was used to calculate the time frames of the source images and to consider different peak latency variations among the VM patients (66–102 ms) and HC subjects (86–104 ms). Afterward, non-parametric statistical analyses of sLORETA images (statistical non-parametric mapping; SnPM) were performed to identify differences in source activity generators between VM patients and HC subjects. This was done using sLORETA’s built-in voxel-wise randomization tests with 5000 permutations and employing a log-F-ratio statistic for independent groups with a threshold of *p* < 0.01 (corrected for multiple comparisons).

## Results

### VEPs

**Figure 1:**
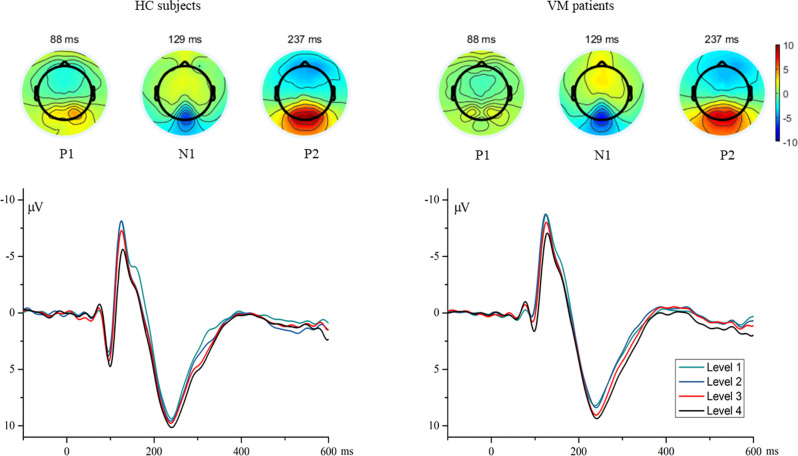
The topographies show the P1 (88 ms), N1 (129 ms), and P2 (237 ms) VEP and are given separately for HC subjects and VM patients. Grand average VEP waveforms recorded at channel Oz are shown for different luminance ratios (Levels 1–4). Levels 1 and 4 refer to the smallest (12.5%) and largest (50%) proportions of white pixels in the stimulus pattern, respectively. VEP, visual evoked potential; HC, healthy control; VM, vestibular migraine.

Individual waveform averages were averaged together for each group to produce a grand-average waveform and topographic maps of HC subjects and VM patients as shown in Figure 1. The results revealed smaller amplitudes and reduced latencies of the P1 component in VM patients. We compared the amplitudes and latencies of the VEP components of both groups with respect to the four checkerboard images.

**Figure 2:**
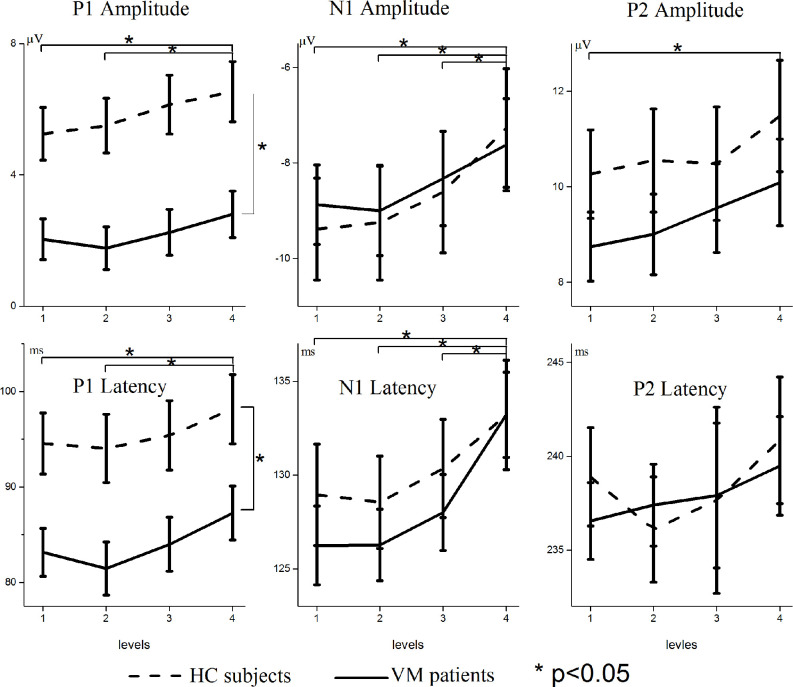
Modulation of scalp-recorded VEPs across different luminance ratios. The amplitudes and latencies of the P1, N1, and P2 VEPs are shown separately for VM patients (solid line) and HC subjects (dashed line). Levels 1 and 4 refer to the smallest (12.5%) and largest (50%) proportions of white pixels in the stimulus pattern, respectively. Asterisks indicate significant differences between VM patients and HC subjects (*p* < 0.05). Note the different scaling for different VEP components.

Figure 2 shows the VEP amplitudes and latencies for different luminance ratios separately for VM patients and HC subjects. Repeated-measures ANOVAs on P1 amplitudes and latencies both revealed significant main effects for condition [*F* = 7.072, *p* = 0.001; *F* = 9.871, *p* = 0.000] and group [*F* = 12.127, *p* = 0.001; *F* = 7.267, *p* = 0.009]; there was no significant interaction between condition and group [*F* = 0.637, *p* = 0.542; *F* = 0.289, *p* = 0.736]. Multiple comparisons on VEP amplitudes and latencies in separate conditions showed that Level 1 evoked significantly smaller amplitudes and shorter latencies than Level 4 [*p* = 0.015; *p* = 0.004], and Level 2 evoked significantly smaller amplitudes and shorter latencies than Level 4 [*p* = 0.014; *p* = 0.000].

Repeated-measures ANOVAs on N1 amplitudes and latencies revealed significant main effects for condition [*F* = 12.509, *p* = 0.000; *F* = 21.889, *p* = 0.000]. There was no significant main effect for group [*F* = 0.014, *p* = 0.906; *F* = 0.319, *p* = 0.575] and no significant interaction between condition and group [*F* = 0.672, *p* = 0.534; *F* = 1.167, *p* = 0.313]. Multiple comparisons on VEP amplitudes and latencies in separate conditions showed that Level 1 evoked significantly smaller amplitudes and shorter latencies than Level 4 [*p* = 0.001; *p* = 0.000], Level 2 evoked significantly smaller amplitudes and shorter latencies than Level 4 [*p* = 0.000; *p* = 0.000], and Level 3 evoked significantly smaller amplitudes and shorter latencies than Level 4 [*p* = 0.042; *p* = 0.000].

Repeated-measures ANOVAs on P2 amplitudes and latencies revealed significant main effects for condition [*F* = 4.076, *p* = 0.011; *F* = 3.449, *p* = 0.043], but no significant main effects for group [*F* = 1.067, *p* = 0.306; *F* = 0.314, *p* = 0.577] and no significant interaction between condition and group [*F* = 0.271, *p* = 0.822; *F* = 3.012, *p* = 0.062]. Multiple comparisons on VEP amplitudes and latencies in separate conditions showed that Level 1 stimuli evoked significantly smaller amplitudes than Level 4 [*p* = 0.028].

### sLORETA Group Comparisons

**Figure 3:**
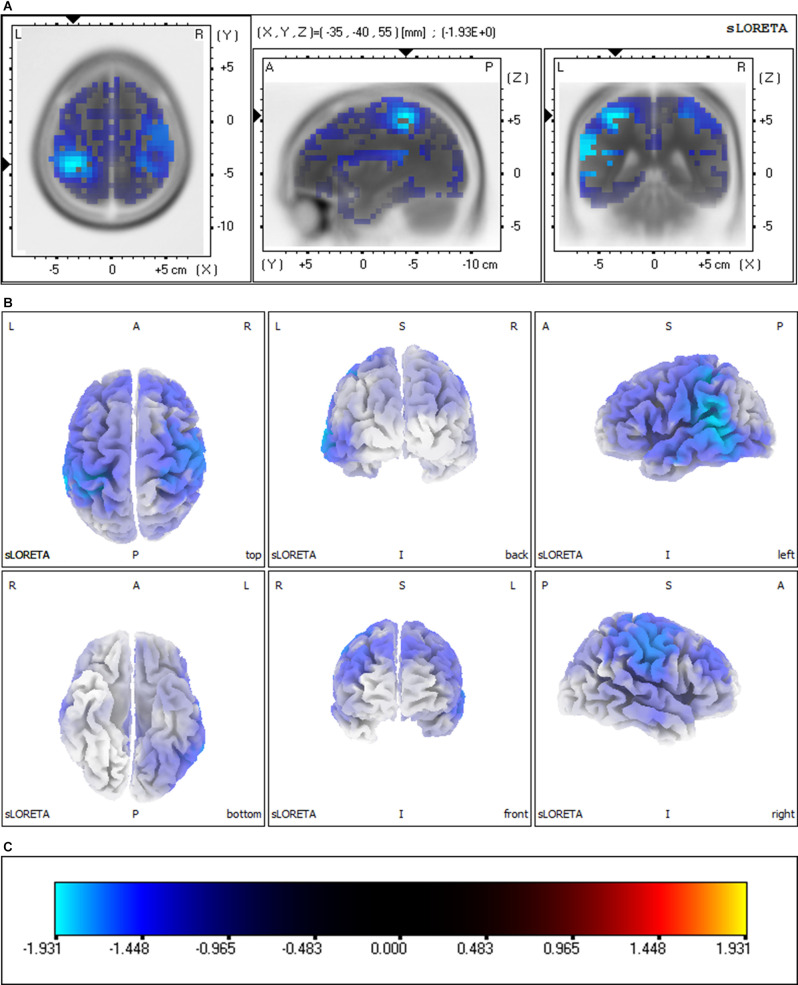
Significant group comparisons of the sLORETA source imaging between the VM and HC groups. Regions with significant differences between groups are shown in three MRI views of the head **(A)** and 3D brain map views **(B)**. The color scale **(C)** represents log-F ratio values (threshold: log-F = 0.836, *p* < 0.01, two-tailed). The difference in current density maximum was highest in the postcentral gyrus of the parietal lobe [MNI coordinates (x, y, z = −35, −40, 55), BA 40; logF = −1.93, *p* < 0.001]. L, left; R, right; A, anterior; P, posterior; BA, Brodmann area.

Group comparisons of the sLORETA source imaging are shown in Figure 3. Locations with significant differences between VM patients and HC subjects are shown in two different forms: MRI views of three sides of the brain (Figure 3A) and 3D brain maps (Figure 3B). These maps and figures were created by assigning the SnPM t-values (two-tailed threshold) to their corresponding BAs, and color-coded (Figure 3C) using a range of light blue over dark blue, black, and red to yellow. Light blue represents negative t-values, which shows that the current source density of this location has significantly decreased. Compared with the P1 source densities of HC subjects, significant current density decrements (threshold log-F-ratio = 0.836, *p* < 0.01) of VM patients were distributed over the frontal lobe, parietal lobe, temporal lobe, limbic lobe, occipital lobe, and sub-lobar regions. Table 2 provides a complete overview of all retrieved statistically significant results including all anatomical regions and the number of activated voxels. The difference in the current density maximum was highest in the postcentral gyrus of the parietal lobe [Montreal Neurological Institute (MNI) coordinates (x, y, *z* = −35, −40, 55), BA 40] (logF = −1.93, *p* < 0.001).

**Table 2:** Significant brain regions of P1 source activities and the numbers of voxels differing between VM patients and HC subjects.

Lobe	L	R	Total
**Frontal lobe**	**994**	**889**	**1,883**
Middle frontal gyrus	219	230	449
Precentral gyrus	177	180	357
Inferior frontal gyrus	176	150	326
Superior frontal gyrus	151	130	281
Medial frontal gyrus	151	123	274
Paracentral lobule	50	22	72
Rectal gyrus	21	22	43
Orbital gyrus	15	9	24
Subcallosal gyrus	14	9	23
Sub-gyral	9	7	16
Cingulate gyrus	6	4	10
Postcentral gyrus	4	2	6
Precuneus	1	1	2
**Parietal lobe**	**582**	**547**	**1,129**
Postcentral gyrus	175	178	353
Precuneus	160	132	292
Inferior parietal lobule	142	142	284
Superior parietal lobule	63	63	126
Supramarginal gyrus	20	15	35
Angular gyrus	11	7	18
Sub-gyral	9	8	17
Paracentral lobule	2	2	4
**Temporal lobe**	**568**	**403**	**971**
Superior temporal gyrus	199	173	372
Middle temporal gyrus	165	144	309
Inferior temporal gyrus	74	35	109
Fusiform gyrus	85	15	100
Transverse temporal gyrus	18	18	36
Sub-gyral	12	8	20
Supramarginal gyrus	8	4	12
Angular gyrus	5	4	9
Inferior frontal gyrus	1	1	2
Insula	1	1	2
**Limbic lobe**	**368**	**226**	**594**
Cingulate gyrus	143	119	262
Anterior cingulate	65	45	110
Parahippocampal gyrus	85	21	106
Posterior cingulate	38	21	59
Uncus	31	15	46
Precuneus	2	3	5
Sub-gyral	2	1	3
Paracentral lobule	1	1	2
Inferior temporal gyrus	1		1
**Occipital lobe**	**337**	**132**	**469**
Cuneus	124	86	210
Middle occipital gyrus	67	16	83
Lingual gyrus	65		65
Precuneus	21	18	39
Fusiform gyrus	29		29
Inferior occipital gyrus	16	2	18
Superior occipital gyrus	7	6	13
Middle temporal gyrus	6	2	8
Inferior temporal gyrus	2	2	4
**Sub-lobar**	**111**	**106**	**217**
Insula	104	101	205
Extra-nuclear	7	5	12

## Discussion

**Figure 4:**
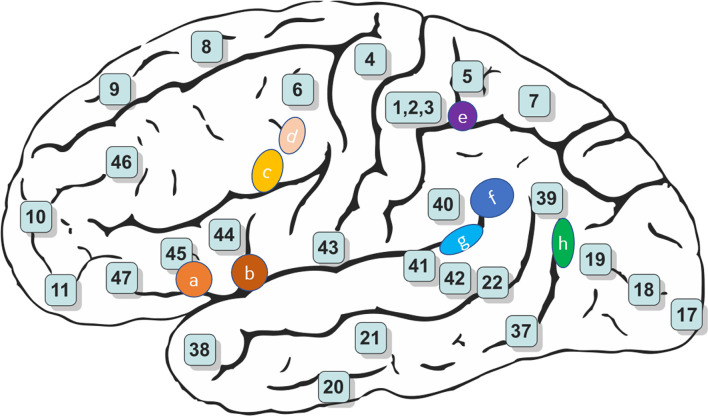
Schematic brain representations illustrating the topography of the vestibular cortical fields experimentally identified in humans. The numbers indicate the architectonically defined BAs [based on Gray’s (1918) Anatomy of the Human Body]. The letters represent the vestibular sites with their localization in the cortical regions in the right panel (Ventre-Dominey, 2014).

The present study examined VEP responses in patients with VM and HC subjects. For the P1 VEP, our results revealed significantly lower amplitude and decreased latency, and reduced cortical activation in VM patients compared with HC subjects. The mechanisms underlying abnormal VEP responses to visual stimulation in patients with VM are currently unknown, but we know that the P1 source density differences between HC subjects and VM patients overlapped with the vestibular cortical fields (Figures 3, 4).

We found reduced P1 amplitude and shorter P1 latency for VM patients compared with HC subjects, which indicates that VM is associated with functional changes in the central visual system. This observation may be explained by a lowered cortical preactivation level or reduced baseline activation of sensory cortices leading to abnormal information processing. sLORETA analysis was used to identify which brain regions contributed to these alterations. We identified reduced source activation of P1 in the frontal, parietal, temporal, limbic, and occipital lobes and sub-lobar regions in VM patients compared with HC subjects, which is in line with previous functional magnetic resonance imaging (fMRI) studies that showed cortical gray matter changes in vestibular migraine (Zhe et al., 2021). Vestibular-sensitive neurons respond to a variety of modalities including proprioception or visual motion. Vestibular information and somatosensory and/or visual inputs are conveyed through ventral posterolateral, ventral posteromedial, and ventral posterior inferior thalamic nuclei and lateral geniculate nucleus. We found that the P1 source density differences between HC subjects and VM patients overlapped with the vestibular cortical fields. This led us to hypothesize deactivation of the vestibular cortex regions in VM patients, where excessive inhibition of the vestibular cortex regions leads to peripheral loss of control, resulting in peripheral dysfunction such as photophobia and vestibular dysfunction.

Vestibular information processing involves polymodal association areas in the parietal, temporal, and insular cortices and cortical areas associated with spatial orientation (e.g., primary somatosensory cortex, primary visual cortex; Vuralli et al., 2018). When visual, vestibular, and somatosensory stimuli incorporation and/or processing of one of these three components is damaged, VM victims may experience vestibular symptoms. We hypothesized a central disruption in multisensory component processing, though how this would affect visual or vestibular system processing remains unknown in VM.

Few studies have thoroughly examined VEPs at different luminance ratios in VM patients. Our results indicated parametric regulation of P1 amplitudes based on the luminance ratio in the checkerboard images. This result was found in both VM patients and HC subjects and is in accordance with previous findings of larger VEP reactions for higher luminance levels in visual stimuli (Johannes et al., 1995; Sandmann et al., 2012). Further topographic mapping analysis revealed that VM patients and HC subjects had a P1 latency difference in the occipital area, which corroborates the previous finding that reduced metabolism in the occipital cortex may signify mutual suppression between the visual and vestibular systems (Shin et al., 2014).

Compared with HC subjects, we found that VM patients showed considerably shorter P1 latencies. As VEP latencies can be used to approximate visual processing time (Thorpe et al., 1996) and might change with latency in the behavioral reaction in visual tasks (Fort et al., 2005), we predict that this finding may reveal quicker, more effective visual information processing that enables faster behavioral reactions in these subjects. However, while increased sensitivity to visual stimuli might be linked to the quicker low-level visual processing observed in migraineurs with aura (Wray et al., 1995), it is not clear whether patients with VM exhibit enhanced (behavioral) response speed in visual tasks compared with HC subjects. The fact that the VM group showed marked response time advantages in basic tasks provides psychophysical validation of their expected hypersensitivity to visual stimuli. These results suggest that since signals to the primary visual cortex are processed more promptly, VM patients are faster at low-level visual processing.

VM patient processing speed for low-level visual tasks should perhaps be seen as reflecting an interaction between more or less visual excitation and more or less regional inhibitory failure (Wray et al., 1995). To test this hypothesis, future research should combine electrophysiological recordings with neuroimaging studies so that the temporal patterns of sensory processing can be correlated with the accompanying anatomical and functional changes in patients with VM.

## Conclusions

Using a combined VEP and sLORETA approach, we found smaller P1 amplitudes and decreased latency, and reduced visual cortex activation in VM patients compared with HC subjects. These findings suggest altered processing of visual stimuli in VM patients between attacks. Specifically, we found that the P1 source density differences between HC subjects and VM patients overlap with the vestibular cortical fields. These results suggest that abnormalities in vestibular cortical fields might be a pathophysiological mechanism of VM.

## Data Availability Statement

The original contributions presented in the study are included in the article, further inquiries can be directed to the corresponding author/s.

## Ethics Statement

The studies involving human participants and the protocol were reviewed and approved by the Ethics Committee of Sun Yat-sen University. The patients/participants provided their written informed consent to participate in this study.

## Author Contributions

YO designed the experiment and revised the manuscript. JLiu and ML performed the experiment, interpreted the results, and wrote the manuscript. QZ and YW improved the experiment and the article. YCh, JW, and JLi helped to analyze the data. LC, LY, and YCa helped to collect patient. YZ supervised the work. All authors read and approved the final manuscript. All authors contributed to the article and approved the submitted version.

## Conflict of Interest

The authors declare that the research was conducted in the absence of any commercial or financial relationships that could be construed as a potential conflict of interest.

## Publisher’s Note

All claims expressed in this article are solely those of the authors and do not necessarily represent those of their affiliated organizations, or those of the publisher, the editors and the reviewers. Any product that may be evaluated in this article, or claim that may be made by its manufacturer, is not guaranteed or endorsed by the publisher.

## Footnotes

^1^ https://www.uzh.ch/keyinst/NewLORETA/Software/Software.htm
